# A review of estimation of distribution algorithms in bioinformatics

**DOI:** 10.1186/1756-0381-1-6

**Published:** 2008-09-11

**Authors:** Rubén Armañanzas, Iñaki Inza, Roberto Santana, Yvan Saeys, Jose Luis Flores, Jose Antonio Lozano, Yves Van de Peer, Rosa Blanco, Víctor Robles, Concha Bielza, Pedro Larrañaga

**Affiliations:** 1 Department of Computer Science and Artificial Intelligence, University of the Basque Country, Donostia – San Sebastián, Spain; 2 Department of Plant Systems Biology, Ghent University, Ghent, Belgium; 3 Department of Molecular Genetics, Ghent University, Ghent, Belgium; 4 Department of Statistics and Operations Research, Public University of Navarre, Pamplona, Spain; 5 Departamento de Arquitectura y Tecnología de Sistemas Informáticos, Universidad Politécnica de Madrid, Madrid, Spain; 6 Departamento de Inteligencia Artificial, Universidad Politécnica de Madrid, Madrid, Spain

## Abstract

Evolutionary search algorithms have become an essential asset in the algorithmic toolbox for solving high-dimensional optimization problems in across a broad range of bioinformatics problems. Genetic algorithms, the most well-known and representative evolutionary search technique, have been the subject of the major part of such applications. Estimation of distribution algorithms (EDAs) offer a novel evolutionary paradigm that constitutes a natural and attractive alternative to genetic algorithms. They make use of a probabilistic model, learnt from the promising solutions, to guide the search process. In this paper, we set out a basic taxonomy of EDA techniques, underlining the nature and complexity of the probabilistic model of each EDA variant. We review a set of innovative works that make use of EDA techniques to solve challenging bioinformatics problems, emphasizing the EDA paradigm's potential for further research in this domain.

## Introduction

As a consequence of increased computational power in the last decades, evolutionary search algorithms emerged as important heuristic optimization techniques in the early eighties. Evolutionary optimization techniques have demonstrated their potential across a broad spectrum of areas such as transportation, machine learning or industry. Based on the development of current high-throughput data capturing devices in biotechnology, a wide range of high-dimensional optimization problems surfaced in the field of bioinformatics and computational biology over the last decade. Because classic optimization techniques only explore a limited portion of the solution space, researchers soon realized that sequential search engines that try to improve a single solution are clearly insufficient to move through these huge search spaces. The use of population-based, randomized search engines was proposed as an alternative that would overcome these limitations and be better able to explore the vast solution space. Evolutionary optimization techniques, of which genetic algorithms (GAs) are the most well known class of techniques, have thus been the method of choice for many of these bioinformatics problems.

Estimation of distribution algorithms (EDAs) are a novel class of evolutionary optimization algorithms that were developed as a natural alternative to genetic algorithms in the last decade. The principal advantages of EDAs over genetic algorithms are the absence of multiple parameters to be tuned (e.g. crossover and mutation probabilities) and the expressiveness and transparency of the probabilistic model that guides the search process. In addition, EDAs have been proven to be better suited to some applications than GAs, while achieving competitive and robust results in the majority of tackled problems. In this review, we focus on a group of pioneering papers that have shown the power of the EDA paradigm in a set of recent bioinformatic, mainly genomic and proteomic, tasks. For each problem, we give a brief description, the EDA used, and the associated literature references. The solution representation and the cardinality of the search space are also discussed in some cases. Before discussing these problems, the next section presents what an EDA is and how it works, sets out a detailed taxonomy based on their main features and what potential they have within the bioinformatic discipline.

## Estimation of distribution algorithms

**Figure 1:**
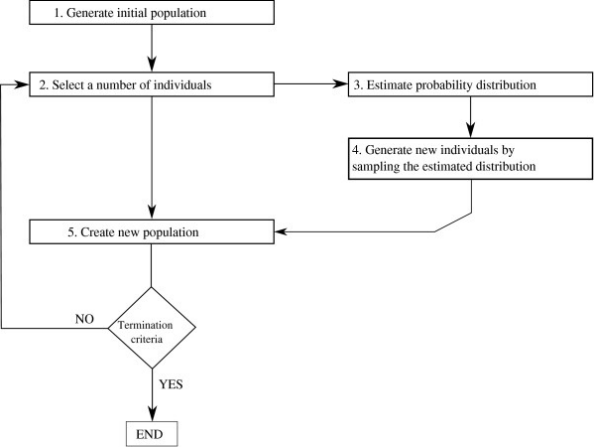
**EDA algorithm flow chart (Figure 1-EDAChart.eps)**. Diagram of how an estimation of distribution algorithm works. This overview of the algorithm is further specified by the pseudocode shown in Table 1.

Estimation of distribution algorithms [1-5] are evolutionary algorithms that work with a multiset (or population sets) of candidate solutions (points). Figure 1 illustrates the flow chart for any EDA approach. Initially, a random sample of points is generated. These points are evaluated using an objective function. An objective function evaluates how accurate each solution is for the problem. Based on this evaluation, a subset of points is selected. Hence, points with better function values have a bigger chance of being selected.

Then, a probabilistic model of the selected solutions is built, and a new set of points is sampled from the model. The process is iterated until the optimum has been found or another termination criterion is fulfilled.

For more details, Table 1 sets out the pseudocode that implements a basic EDA. The reader can find a complete running example of an EDA in [6].

**Table 1:** EDA pseudocode

Set *t *← 0. Generate *M *points randomly
**Do**
Evaluate the points using the fitness function
Select a set *S *of *N *≤ *M *points according to a selection method
Estimate a probabilistic model for *S*
Generate *M *new points sampling from the distribution represented in the model
*t *← *t *+ 1
**until **Termination criteria are met

Estimation of distribution algorithms: evolutionary computation based on learning and simulation of probabilistic graphical models.

### Characteristics of EDAs

Essentially EDAs assume that it is possible to build a model of the promising areas of the search space, and use this model to guide the search for the optimum. In EDAs, modeling is achieved by building a probabilistic graphical model that represents a condensed representation of the features shared by the selected solutions. Such a model can capture different patterns of interactions between subsets of the problem variables, and can conveniently use this knowledge to sample new solutions.

Probabilistic modeling gives EDAs an advantage over other evolutionary algorithms that do not employ models, such as GAs. These algorithms are generally unable to deal with problems where there are important interactions among the problems' components. This, together with EDAs' capacity to solve different types of problems in a robust and scalable manner [3,5], has led to EDAs sometimes also being referred to as competent GAs [7,8].

### A taxonomy of EDAs

Since several EDAs have been proposed with a variety of models and learning algorithms, the selection of the best EDA to deal with a given optimization problem is not always straightforward. One criterion that could be followed in this choice is to trade off the complexity of the probabilistic model against the computational cost of storing and learning the selected model. Both issues are also related to the problem dimensionality (i.e. number of variables) and to the type of representation (e.g. discrete, continuous, mixed).

Researchers should be aware that simple models generally have minimal storage requirements, and are easy to learn. However, they have a limited capacity to represent higher-order interactions. On the other hand, more complex models, which are able to represent more involved relationships, may require sophisticated data structures and costly learning algorithms. The impact that the choice between simple and more complex models has in the search efficiency will depend on the addressed optimization problem. In some cases, a simple model can help to reach non-optimal but acceptable solutions in a short time. In other situations, e.g. deceptive problems, an EDA that uses a simple model could move the search away from the area of promising solutions.

Another criterion that should be taken into consideration to choose an EDA is whether there is any previous knowledge about the problem structure, and which kind of probabilistic model is best suited to represent this knowledge. The following classification of EDAs is intended to help the bioinformatic researcher to find a suitable algorithm for his or her application.

EDAs can be broadly divided according to the complexity of the probabilistic models used to capture the interdependencies between the variables: univariate, bivariate or multivariate approaches. Univariate EDAs, such as PBIL [9], cGA [10] and UMDA [4], assume that all variables are independent and factorize the joint probability of the selected points as a product of univariate marginal probabilities. Consequently, these algorithms are the simplest EDAs and have also been applied to problems with continuous representation [11].

The bivariate models can represent low order dependencies between the variables and be learnt using fast algorithms. MIMIC [12], the bivariate marginal distribution algorithm BMDA [13], dependency tree-based EDAs [14] and the tree-based estimation of distribution algorithm (Tree-EDA) [15] are all members of this subclass. The latter two use tree and forest-based factorizations, respectively. They are recommended for problems with a high cardinality of the variables and where interactions are known to play an important role. Trees and forests can also be combined to represent higher-order interactions using models based on mixtures of distributions [15].

**Figure 2:**
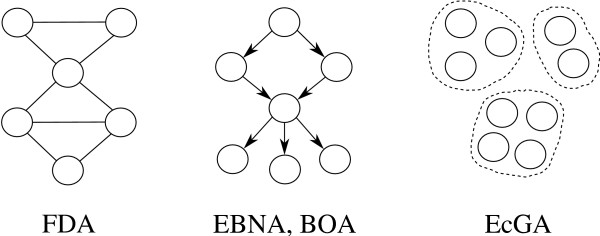
**EBNA and BOA paradigms (Figure 2-EBNA-BOA.eps)**. Diagram of probability models for the proposed EDAs in combinatorial optimization with multiple dependencies (FDA, EBNA, BOA, and EcGA).

Multivariate EDAs factorize the joint probability distribution using statistics of order greater than two. Figure 2 shows some of the different probabilistic graphical models covered by this category. As the number of dependencies among the variables is higher than in the above categories, the complexity of the probabilistic structure, as well as the computational effort required to find the structure that best suits the selected points, is greater. Therefore, these approaches require a more complex learning process. Some of the EDA approaches based on multiply connected Bayesian networks are:

• The (Factorized Distribution Algorithm) FDA [16] is applied to additively decomposed functions for which, using the running intersection property, a factorization of the mass-probability based on residuals and separators is obtained.

• In [17], a factorization of the joint probability distribution encoded by a Bayesian network is learnt from the selected set in every generation. The estimation of Bayesian network algorithm (EBNA) uses the Bayesian information criterion (BIC) score as the quality measure for the Bayesian network structure. The space of models is searched using a greedy algorithm.

• The Bayesian optimization algorithm (BOA) [18] is also based on the use of Bayesian networks. The Bayesian Dirichlet equivalent metric is drawn on to measure the goodness of every structure. The algorithm enacts a greedy search procedure. BOA has been improved by adding dependency trees and restricted tournament replacement. The resulting, more advanced, hierarchical BOA (hBOA) [5] is one of the EDAs for which extensive experimentation has been undertaken. The results show good scalability behavior.

• The extended compact Genetic Algorithm (EcGA) proposed in [10] is an algorithm in which the basic idea is to factorize the joint probability distribution as a product of marginal distributions of variable size.

There are alternatives to the use of Bayesian networks for representing higher order interactions in EDAs. Markov network-based EDAs [19-21] could be an appropriate choice for applications where the structure of the optimization problem is known and can be easily represented using an undirected graphical model. EDAs that use dependency networks [22] can encode dependencies that Bayesian networks cannot represent. Both classes of algorithms need relatively complex sampling procedures based on the use of Gibbs sampling [23].

In addition to the order of complexity encoded by the probability model, there is another key feature when dealing with an EDA algorithm: the way that model is learned. There are two alternatives: induce the model structure and its associated parameters, or induce just the set of parameters for *an a priori *given model. The first class is denoted as *structure*+*parameter learning*, whereas the second is known as *parameter learning*. Both approaches need to induce the parameters of their models, but the first approach's need for structural learning makes it more time consuming. By contrast, parameter learning is dependent on the fixed model, whereas structure+parameter learning exhibits a greater power of generalization.

Population-based incremental learning (PBIL) [9], the compact GA (cGA) [10], the univariate marginal distribution algorithm (UMDA) [4] and the factorized distribution algorithm (FDA) [16] which use a fixed model of interactions in all generations, are all parameter approaches. On the other hand, the mutual information maximization for input clustering algorithm (MIMIC) [12], the extended compact GA (EcGA) [10] and EDAs that use Bayesian and Gaussian networks [5,13,17,24-26] belong to the structural+parameter class.

So as to have a graphical taxonomy of the subdivisions presented through this section, Table 2 illustrates all the above features and models providing a graphical taxonomy of the subdivisions presented throughout this section. It also includes some useful tips to choose among the available EDAs, such as their pros and cons.

**Table 2:** EDAs taxonomy

Statistical order	Advantages	Disadvantages	Examples
**Univariate**	Simplest and fastest	Ignore feature dependencies	PBIL (Baluja, 1994)
	Suited for high cardinality problems	Bad performance for deceptive problems	UMDA (Mühlenbein and Paaß, 1996)
	Scalable		cGA (Harik *et al*., 1999)

**Bivariate **(statistics of order two)	Able to represent low order dependencies	Possibly ignore some feature dependencies	MIMIC (De Bonet *et al*., 1996)
	Suited for many problems	Slower than univariate EDAs	Dependency trees EDA (Baluja and Davies, 1997) BMDA (Pelikan and Mühlenbein, 1999)
	Graphically inquire the induced models		Tree-EDA/Mixture of distributions EDA (Santana *et al*., 1999)

**Multivariate **(statistics of order greater than two)	Parameter learning (*only interaction model parameters*)		
	
	Suited for problems with known underlying model	Possibly ignore complex feature dependencies	FDA (Mühlenbein *et al*., 1999)
		Higher memory requirements than bivariate	Markov network-based EDA (Shakya and McCall, 2007)
	Structure+parameter learning (*interaction model & parameters of the model*)		
	
	Maximum power of generalization	Highest computation time	EcGA (Harik *et al*., 1999)
	Flexibility to introduce user dependencies	Highest memory requirements	EBNA (Etxeberria and Larrañaga, 1999)
	Online study of the induced dependencies		BOA/hBOA (Pelikan *et al*., 1999, 2005)
			Dependency networks EDA (Gámez *et al*., 2007)

A taxonomy of some representative EDAs. We highlight a set of characteristics that can guide the choice of a particular EDA suited to the goals and properties of a given problem.

### Potential of EDAs in bioinformatics

Evolutionary algorithms, and GAs in particular, have been widely and successfully applied in bioinformatics. It is reasonable to expect that the improvements in EDA efficiency and scalability can contribute to expanding the use of these algorithms, particularly for difficult problems where other evolutionary algorithms fail [3,27].

There are other situations where the use of EDAs can be very useful for solving bioinformatics problems. For instance, probabilistic models used by EDAs can be set up a *priori *in such a way that they represent previous knowledge about the structure of the optimization problem. Even the use of incomplete or partial information about the problem domain can considerably reduce the computational cost of the search. Similarly, practitioners can manipulate the probabilistic models to favor solutions with certain pre-established partial configurations. This way they can test particular hypotheses about the configuration of the optimal solution.

EDAs have another advantage, also associated with the capacity to model key features of the search space. The models generated during the search can be mined to reveal previously unknown information about the problem [28-30].

Furthermore, recent results of applying EDAs to problems from other domains [31] have shown that the information gathered by the models to solve a given problem instance can, in some cases, also be employed to solve other instances of the same problem. This paves the way for building bioinformatics applications where the information extracted from previous searches is reused to solve different instances of a similar problem.

## EDAs in genomics

### Introduction

Due to advances in modern high-throughput biotechnology devices, large and high-dimensional data sets are obtained from analyzed genomes and tissues. The heuristic scheme provided by EDAs has proved to be effective and efficient, in a variety of NP-hard genomic problems. Because of the huge cardinality of the solution spaces of most of these problems, researchers are aware of the need for an efficient optimization algorithm. In this way, authors have preferred simple EDA schemes that assume that the variables are independent. These schemes have obtained accurate and robust solutions in reasonable CPU times. Together with a brief definition of each tackled genomic problem, we describe the main characteristics of each EDA scheme are described, with a special emphasis on the codification used to represent the search individuals.

### Gene structure analysis

As genomes are being sequenced at an increasing pace, the need for automatic procedures for annotating new genomes is becoming more and more important. A first and important step in the annotation of a new genome is the location of the genes in the genome, as well as their correct structure. As a gene may contain many different parts, the problem of gene structure prediction can be seen as a segmentation or parsing problem. To solve this problem automatically, pattern recognition and machine learning techniques are often used to build a model of what a gene looks like. This model can then be used to automatically locate potential genes in a genome [32,33].

A gene prediction framework consists of different components, where each component (often modeled as a classifier) aims at identifying a particular structural element of the gene. Important structural elements include the start of the gene (start codon), the end of a gene (stop codon) and the transitions between the coding and non-coding parts of the gene (splice sites).

The exact mechanisms that the cell uses to recognize genes and their structural elements are still under research. As this knowledge is missing, one major problem in this context is to define adequate features to train the classifiers for each structural element. Consequently, large sets of sequence features are extracted in the hope that these sets will contain the key features. However, it is known that not all of these features will be important for the classification task at hand, and many will be irrelevant or redundant.

To find the most relevant features for recognizing gene structural elements, feature subset selection (FSS) techniques can be used. These techniques try to select a subset of relevant features from the original set of features [34,35]. As this is an NP-hard optimization problem with 2^*n *^possible subsets for evaluation (given n features), population-based heuristic search methods are an interesting engine for driving the search through the space of possible feature subsets. Each solution in the population decodes a feature subset as a binary string: features having a value of 1 are included in the subset, whereas the ones having a value of 0 are discarded.

As a natural alternative to genetic algorithms, the use of EDAs for FSS was initiated in [36] for classic benchmark problems, and their use in large scale feature subset selection domains was reported to yield good results [37,38]. Furthermore, the EDA-based approach to FSS was shown to generalize to feature weighting, ranking and selection [39]. This has the advantage of getting more insight into the relevance of each feature separately, focusing on strongly relevant, weakly relevant, and irrelevant features.

The application of EDA-based FSS techniques in gene structure prediction was pioneered for the most important gene prediction components in [40]. Its most important application was the recognition of splice sites. Using naïve Bayes classifiers, support vector machines and C4.5 decision trees as base classifiers, an UMDA-based FSS scheme was used to obtain higher performance models.

In addition to better models, an UMDA-based approach was also used to get more insight into the selected features. This led to both the identification of new characteristics, as well as the confirmation of important previously known characteristics [41].

### Gene expression analysis

The quantitative and qualitative DNA analysis is one of the most important areas of modern biomedical research. DNA microarrays can simultaneously measure the expression level or activity level of thousands of genes under a set of conditions. Microarray technology has become a popular option for partial DNA analysis since Golub *et al*.'s pioneering work [42].

The starting point of this analysis is the so called gene expression matrix, where rows represent genes, columns represent experimental conditions (or samples), and the values at each position of the matrix characterize the expression level of the particular gene under the particular experimental condition. Additional biological information about the genes and the experimental conditions can be added to the matrix in the form of gene and/or sample annotation. Depending on how we treat the annotation, gene expression data analysis can be either supervised or unsupervised. When sample annotation is used to split the set of samples into two or more classes or phenotypes (e.g. 'healthy' or 'diseased' tissues), supervised analysis (or class prediction) tries to find patterns that are characteristic of each of the classes. On the other hand, unsupervised analysis (or class discovery) ignores any annotation. Examples of such analysis are gene clustering, sample clustering and gene expression data biclustering.

The FSS paradigm has taken a leading role due to the challenge posed by the huge dimension of DNA microarray studies (datasets of close to 20,000 genes can be found in the experimental setups reported in recent literature), small sample sizes (gene expression studies with more than a hundred hybridizations are not common) and the notable influence of different sources of noise and variability. Thus, the application of dimensionality reduction techniques has become a must for any gene expression analysis.

#### Classification of DNA microarray data

It is broadly assumed that a limited number of genes can cause the onset of a disease. Within this scenario biologists demand a reduction in the number of genes. In addition, the application of a FSS technique to microarray datasets is an essential step to achieve an accurate classification performance for any base classifier.

Although univariate gene ranking procedures are very popular for differential gene expression detection, the multivariate selection of a subset of relevant and non-redundant genes has borrowed from the field of heuristic search engines to guide the exploration of the huge solution space (there are 2^*n *^possible gene subsets, where *n *is the number of initial genes). Two research groups have proven that the EDA paradigm is useful for this challenging problem. Both groups have implemented efficient algorithms that have achieved accuracy levels comparable to the most effective state-of-the-art optimization techniques:

• Using a naïve Bayes network as the base classifier and the UMDA as the search algorithm, Blanco *et al*. [43] achieve competitive results in two gene expression benchmarking datasets. The authors show that the predictive power of the models can be improved when the probability of each gene being selected in the first population is initialized using the results provided by a set of simple sequential search procedures.

• Paul and Iba [44,45] propose two variations of the PBIL search algorithm to identify subsets of relevant and non-redundant genes. Using a wide variety of classifiers, notable results are achieved in a set of gene expression benchmarking datasets with subsets of extremely low dimensionality.

Using a continuous-value version of the UMDA procedure, EDAs have been used as a new way of regularizing the logistic regression model for microarray classification problems [46]. Regularization consists of shrinking the parameter estimates to avoid their unstability present when there are a huge number of variables compared to a small number of observations (as in the microarray setting). Therefore, the parameter estimators are restricted maximum likelihood estimates, i.e. the maximum value of a new function including the likelihood function, plus a penalty term where the size of the estimators is constrained. There are different norms for measuring estimators size. This leads to different regularized logistic regression names [47]: ridge, Lasso, bridge, elastic net, etc.

EDAs could be used to optimize these new functions and be a good optimization method especially in some cases where numerical methods are unable to solve the corresponding non-differentiable and non-convex optimization problems. However, another possibility, taken up in [46], is to use EDAs to maximize the likelihood function without having to be penalized (which is a simpler optimization problem) and to include the shrinkage of the estimates during the simulation of the new population. New estimates are simulated during EDA evolutionary process in such a way that guarantees their shrinkage while maintaining their probabilistic dependence relationships learnt in the previous step. This procedure yields regularized estimates at the end of the process.

#### Clustering of DNA microarray data

Whereas the above papers propose a supervised classification framework, clustering is one of the main tools used to analyze gene expression data obtained from microarray experiments [48]. Grouping together genes with the same behaviour across samples, that is, gene clusters, can suggest new functions for all or some of the grouped genes. We highlight two papers that use EDAs in the context of gene expression profile clustering:

• Peña *et al*. [49] present an application of EDAs for identifying clusters of genes with similar expression profiles across samples using unsupervised Bayesian networks. The technique is based on an UMDA procedure that works in conjunction with the EM clustering algorithm. To evaluate the proposed method, synthetic and real data are analyzed. The experimentation with both types of data provides clusters of genes that may be biologically meaningful and, thus, interesting for biologists to research further.

• Cano *et al*. [50] use UMDA and genetic algorithms to look for clusters of genes with high variance across samples. A real microarray dataset is analyzed, and the Gene Ontology Term Finder is used to evaluate the biological meaning of the resulting clusters.

Like clustering, biclustering is another NP-hard problem that was originally considered by Morgan and Sonquist in 1963 [51]. Biclustering is founded on the fact that not all the genes of a given cluster should be grouped into the same conditions due to their varying biological activity. Thus, biclustering assumes that several genes will only change their expression levels within a specified subset of conditions [52]. This assumption has motivated the development of specific algorithms for biclustering analysis.

An example is the work by Palacios *et al*. [53], which applies an UMDA scheme to search the possible bicluster space. They get accurate results compared to genetic algorithms when seeking single biclusters with coherent evolutions of gene expression values. Like the classic codification discussed for the FSS problem, the authors use two concatenated binary arrays to represent a bicluster, (*x*_1_, ..., *x*_*n *_| *y*_1_, ..., *y*_*m*_). The first array represents each gene of the microarray, where the size is the number of genes. The second array represents each condition, with a size equal to the number of conditions. A value of 1 in the *i*^*th *^position of the first array shows that the *i*^*th *^gene has been selected for inclusion in the bicluster. Likewise, a value of 1 in the *j*^*th *^position of the second array indicates that the *j*^*th *^condition has been selected for inclusion in the bicluster. This codification results in a space of 2^*n*+*m *^possible biclusters.

#### Inference of genetic networks

The inference of gene-gene interactions from gene expression data is a powerful tool for understanding the system behaviour of living organisms [54].

This promising research area is now of much interest for biomedical practitioners, and a few papers have even applied EDAs to this domain. One of these early works uses Bayesian networks as the paradigm for modeling the interactions among genes, while an UMDA approach explores the search space to find the candidate interactions [55]. The subsequent literature evaluation of the most reliable interactions unveils that many of them have been previously reported in the literature.

## EDAs in proteomics

### Introduction

The objective of protein structure prediction is to predict the native structure of a protein from its sequence. In protein design, the goal is to create new proteins that satisfy some given structural or functional constraints. Frequently, both problems are addressed using function optimization. As the possible solution space is usually huge, complex and contains many local optima, heuristic optimization methods are needed. The efficiency of the optimization algorithm plays a crucial role in the process. In this section, we review applications of EDAs to different variants of protein structure prediction and protein design problems.

We start by reviewing some important concepts related to protein models and energy functions in optimization. Then, we propose an initial general classification of EDA applications to protein problems according to how sophisticated and detailed the protein models used are. Subsequently, we give a more detailed classification based on the specificities of the protein problems.

### Protein structure prediction and protein design

Protein structure prediction and protein design are usually addressed by minimizing an energy function in the candidate solution space. Two essential issues in the application of EDAs and other optimization algorithms to these problems are the type of protein representation employed and the energy function of choice.

There are many factors that influence the stability of proteins and have to be taken into account to evaluate candidate structures. The native state is thought to be at the global free energy minimum of the protein. Electrostatic interactions, including hydrogen bonds, van der Waals interactions, intrinsic propensities of the amino acids to take up certain structures, hydrophobic interactions and conformational entropy contribute to free energy. Determining to what extent the function can represent all of these factors, as well as how to weight each one are difficult questions that have to be solved before applying the optimization method.

**Figure 3:**
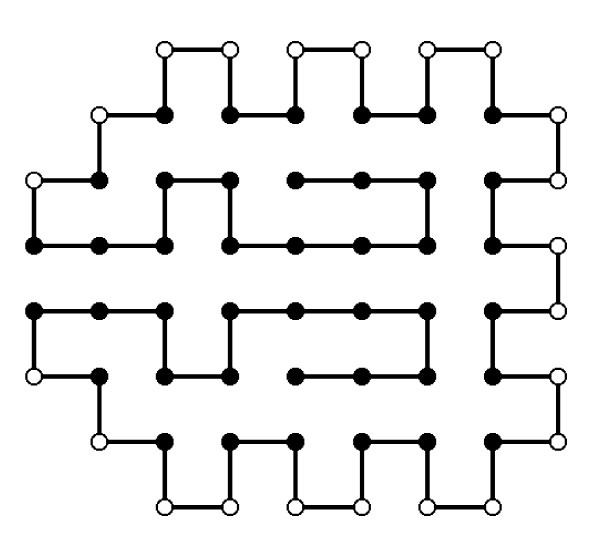
**Optimal protein structure (Figure 3-ProteinStructure.eps)**. Optimal solution of an HP model found by an EDA that uses a Markovian model.

Simplified protein models omit some of these factors and are a first problem-solving approximation. For example, the approximate fold of a protein is influenced by the sequence of hydrophobic and hydrophilic residues, irrespective of what the actual amino acids in that sequence are [56]. Therefore, a first approximation could simply be constructed by a binary patterning of hydrophobic and hydrophilic residues to match the periodicity of secondary structural elements. Simplification can be further developed to consider proteins represented using this binary patterning and to approximate the protein structure prediction problem as two- and three-dimensional lattices. In this case, the energy function measures only hydrophobic and hydrophilic interactions. An example of this type of representation is shown in Figure 3, where a sequence of 64 aminoacids is represented on a two-dimensional lattice.

### EDA approaches

Depending on how sophisticated and detailed the protein model used is, EDAs can be divided into two groups: EDAs applying a simplified model [57-60] and EDAs using more detailed (atomic-based) models [61-63]. A more thorough classification is related to the type of problems addressed:

• Protein structure prediction in simplified models [58,60].

• Protein side chain placement [62,63].

• Design of protein peptide ligands [61].

• Protein design by minimization of contact potentials [59,64].

• Aminoacid alphabet reduction for protein structure prediction [57].

• Using EDAs as a simulation tool to investigate the influence of different protein features in the protein folding process [63].

In [58-60], EDAs are used to solve bi-dimensional and three-dimensional simplified protein folding problems. The hydrophobic-polar (HP) [65], and functional protein models [66] are optimized using EDAs based on probabilistic models of different complexity (i.e. Tree-EDA [67], mixtures of trees EDA (MT-EDA) [67] and EDAs that use *k*-order Markov models (MK-EDA_*k*_) [58]).

The results achieved outperform other evolutionary algorithms. For example, the configuration shown in Figure 3 is the optimal solution found by MK-EDA_2_. Due to the particular topology of this instance, other evolutionary algorithms consistently fail to find the optimal solution [58].

Side chain placement problems are dealt with using UMDA with discrete representation in [62,63]. The approach is based on the use of rotamer libraries that can represent the side chain configurations using their rotamer angles. For these problems, EDAs have achieved very good results in situations where other methods fail [63]. Results are better when EDAs are combined with local optimization methods as in [63], where variable neighborhood search [68] is applied to the best solutions found by UMDA.

Belda *et al*. [61] use different EDAs to generate potential peptide ligands of a given protein by minimizing the docking energy between the candidate peptide ligand and a user-defined area of the target protein surface. The results of the population based incremental learning algorithm (PBIL) [9] and the Bayesian optimization algorithm (BOA) [18] are compared with two different types of genetic algorithms. Results showed that some of the ligands designed using the computational methods had better docking energies than peptides designed using a purely chemical knowledge-based approach [61].

In [64], three different EDAs are applied to solve a protein design problem by minimizing contact potentials: UMDA, Tree-EDA and Tree-EDA^*r *^(the structure of the tree is deduced from the known protein structure, tree parameters are learned from data). Combining probabilistic models able to represent probabilistic dependencies with information about residue interactions in the protein contact graph is shown to improve the search efficiency for the evaluated problems. In [59], EDAs that use loopy probabilistic models are combined with inference-based optimization algorithms to deal with the same problems. For several protein instances, this approach manage to improve the results obtained with tree-based EDAs.

The alphabet reduction problem is addressed in [57] using the extended compact genetic algorithm (EcGA) [69]. The problem is to reduce the 20-letter amino acid (AA) alphabet into a lower cardinality alphabet. A genetics-based machine learning technique uses the reduced alphabet to induce rules for protein structure prediction features. The results showed that it is possible to reduce the size of the alphabet used for prediction from twenty to just three letters resulting in more compact rules.

Results of using EDAs and the HP model to simulate the protein folding process are presented in [64]. Some of the features exhibited by the EDA model that mimics the behaviour of the protein folding process are investigated. The features considered include the correlation between the EDA success rate and the contact order of the protein models, and the relationship between the generation convergence of EDAs for the HP model and the contact order of the optimal solution. Other issues analyzed are the differences in the rate of formation of native contacts during EDA evolution, and how these differences are associated with the contact separation of the protein instance.

## Conclusion

Throughout this paper, we reviewed the state-of-the-art of EDA applications in bioinformatics. As soon as researchers realized the need to apply a randomized, population-based, heuristic search, EDAs emerged as a natural alternative to commonly used genetic algorithms. Since the possible solution space is huge for most of the addressed problems, researchers have made use of efficient EDA implementations.

A group of interesting papers demonstrate the efficiency and the competitive accuracy of this novel search paradigm in a set of challenging NP-hard genomic and proteomic bioinformatic tasks. As the number of EDA application papers in bioinformatics is modest and the number and variety of problems is constantly growing, there is room for new EDA applications in the field.

An interesting opportunity for future research is the adaptation and application of multivariate EDA models that can efficiently deal with the huge dimensionality of current bioinformatic problems. Going further than simple univariate models, bio-experts could explicitly inspect the probabilistic relationships among problem variables for each generation of the evolutionary process. This would create opportunities for improved accuracy. These probabilistic relationships induced from the evolutionary model are an attractive way of proposing novel biological hypotheses to be further tested by bio-experts.

## Competing interests

The authors declare that they have no competing interests.

## Authors' contributions

RA, II, and PL conceived of the manuscript. II, YS, JLF, RB, VR and CB participated in writting the genomics section. The proteomics section was designed and written by RS and JAL. The introduction to EDAs was carried out by RA, RS and YS. RA was in charge of the writing and coordination process. II, YVP and PL helped to write and correct the manuscript draft. All authors read and approved the final manuscript.
